# Supplementary material for: The NOTCH-RIPK4-IRF6-ELOVL4 Axis Suppresses Squamous Cell Carcinoma
Source: Cancers (Basel). 2023 Jan 25;15(3):737. doi: 10.3390/cancers15030737 (PMC9913669; doi:10.3390/cancers15030737)
Supplement: Supplementary file 1 [file cancers-15-00737-s001.zip › Supplementary Figures.pdf]

The NOTCH-RIPK4-IRF6-ELOVL4 axis suppresses squamous cell carcinoma

Supplementary Figures

Authors: Yue Yan[1], Marc-Andre Gauthier[1], Ahmad Malik[2,3], Iosifina Fotiadou[2,3], Michael Ostrovski[2,3], Dzana Dervovic[2], Logine Ghadban[1], Ricky Tsai[2], Gerald Gish[2], Sampath Kumar Loganathan[1,4,5]*, Daniel Schramek[2,3]*

Affiliations:

[1]Department of Otolaryngology, Head and Neck Surgery, Faculty of Medicine, McGill University, Montreal, Canada &

Cancer Research Program, Research Institute of the McGill University Health Centre, Montreal, Canada

[2] Centre for Molecular and Systems Biology, Lunenfeld-Tanenbaum Research Institute, Mount Sinai Hospital, Toronto, Ontario, Canada

[3] Department of Molecular Genetics, University of Toronto, Toronto, Ontario, Canada

[4] Departments of Experimental Surgery and Experimental Medicine, Faculty of Medicine, McGill University, Canada

[5]Rosalind and Morris Goodman Cancer Research Institute, McGill University, Canada

*Corresponding author. Email: schramek@lunenfeld.ca, sampath.loganathan@mcgill.ca

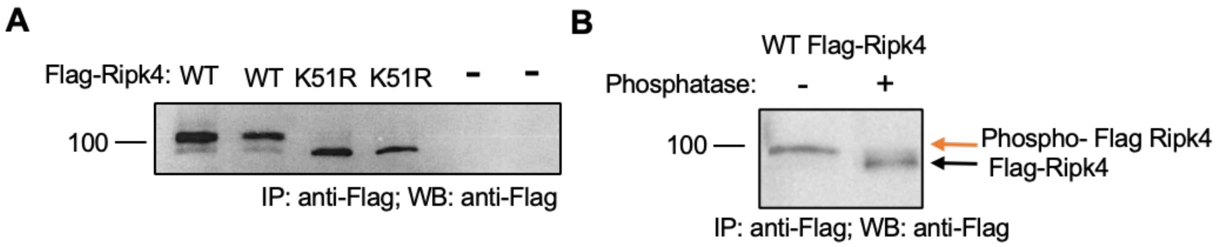

Supplementary Figure 1. Ripk4 tumor suppressor function is dependent on its kinase activity. (**A**) Western blot analysis to test the exogenous expression of Flag-tagged wild type Ripk4 and Ripk4 kinase mutant K51R in mouse keratinocytes. '-' indicates vector only control. (**B**) Western Blot analysis of Flag-tagged WT Ripk4 immuno-precipitated (IP) from mouse keratinocytes and treated with phosphatase or left untreated.

A

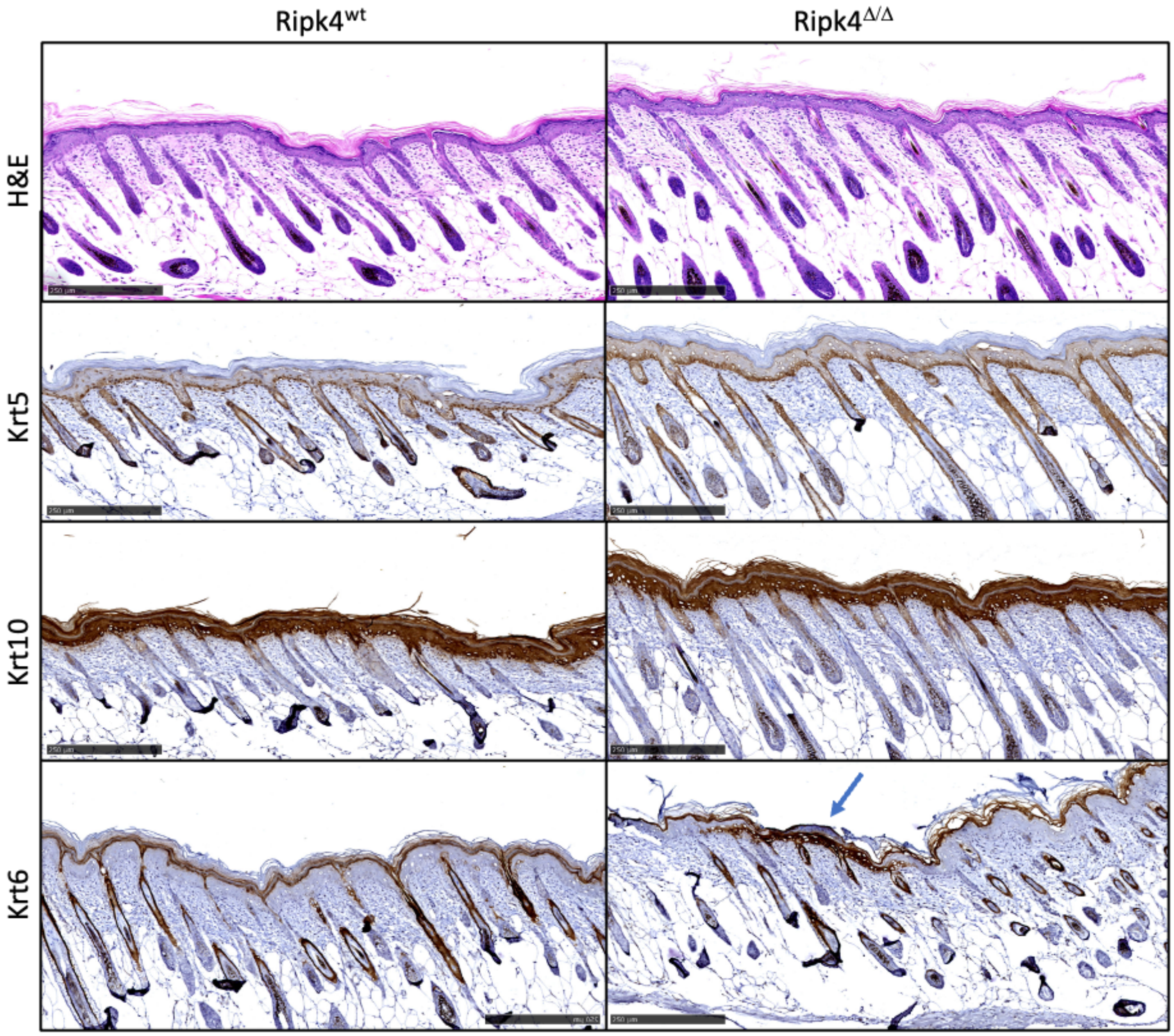

Ripk4^{wt}
Ripk4$^{\Delta/\Delta}$
H&E
Krt5
Krt10
Krt6

B

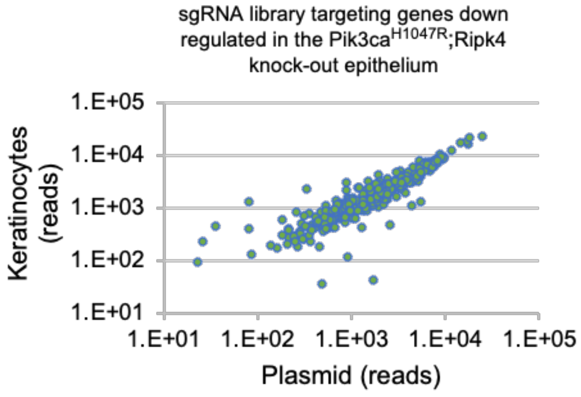

sgRNA library targeting genes down regulated in the Pik3ca^{H1047R};Ripk4 knock-out epithelium
Keratinocytes (reads)
1.E+05
1.E+04
1.E+03
1.E+02
1.E+01
1.E+01
1.E+02
1.E+03
1.E+04
1.E+05
Plasmid (reads)

C

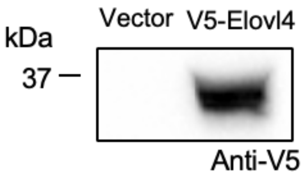

Vector
V5-Elovl4
kDa
37
Anti-V5

D

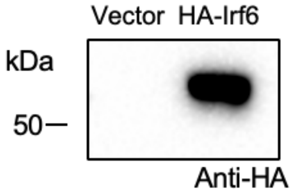

Vector
HA-Irf6
kDa
50
Anti-HA

Supplementary Figure 2. In vivo screen to delineate tumor suppressive Ripk4 target genes

(**A**) Representative H&E images and immunohistochemistry of the indicated markers of P4 skin of *Ripk4*^{wt} and *Ripk4*$^{fl/fl}$ mice transduced with lentivirus (LV) carrying Cre recombinase. Blue arrow indicates a keratin 6 (K6) positive region in *Ripk4*$^{\Delta/\Delta}$ skin. (**B**) Graph showing sgRNA representation from down-regulated genes CRISPR library in plasmid DNA versus epidermal genomic DNA from mice injected in utero with the respective libraries. Each dot represents a guide. Full representation is maintained *in vivo* with some correlation in abundance. (**C and D**) Western Blot analysis of HA-tagged *Irf6* and V5-tagged *Elovl4* in mouse keratinocytes transduced with lenti-virus that were used in Figure 4E. Empty vector was used as a control.

A) Blot for Figure 1C

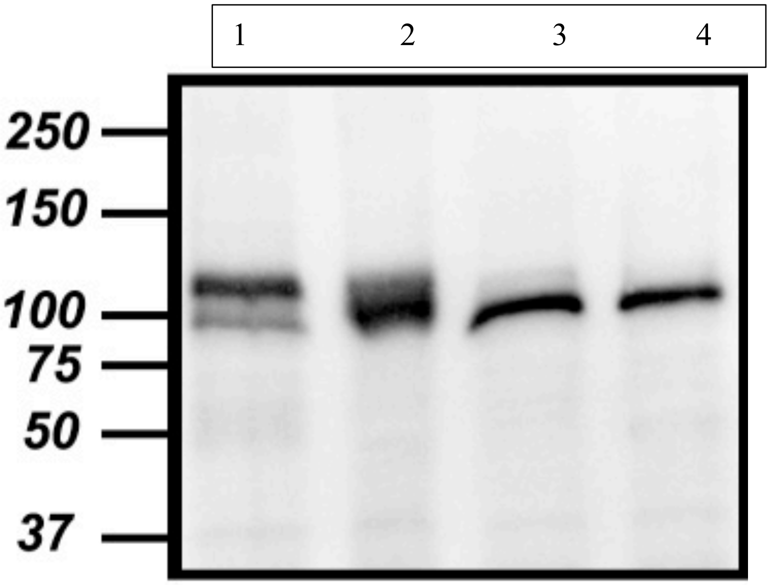

Band at 100 kDa	ROI Intensity
1	11157.024
2	11996.560
3	9787.731
4	6966.569

B) Blot for Supplementary Figure 1A

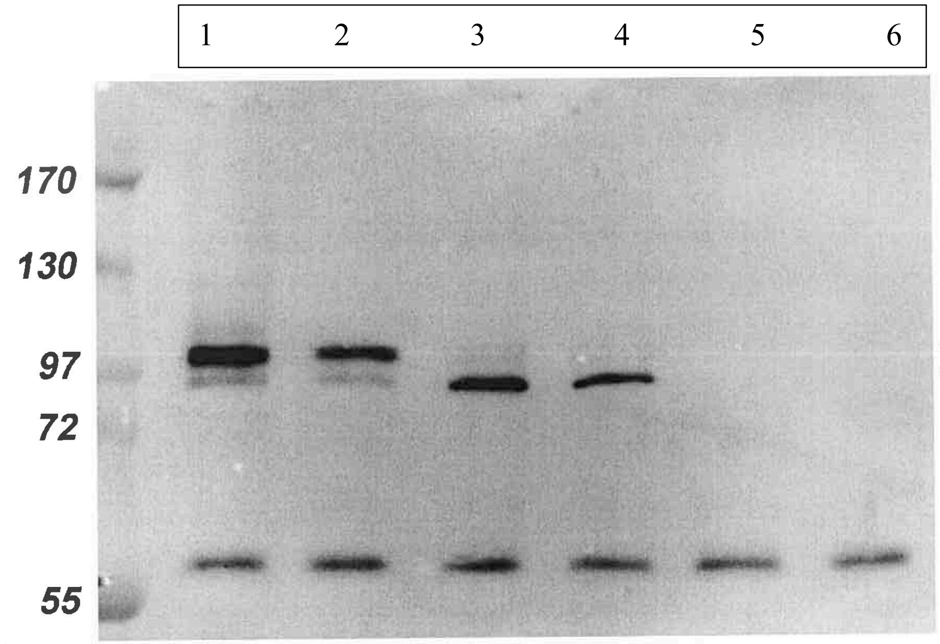

Band at 97 kDa	ROI Intensity
1	7641.610
2	5417.933
3	4496.296
4	3206.447
5	177.971
6	296.092

C) Blot for Supplementary Figure 1B

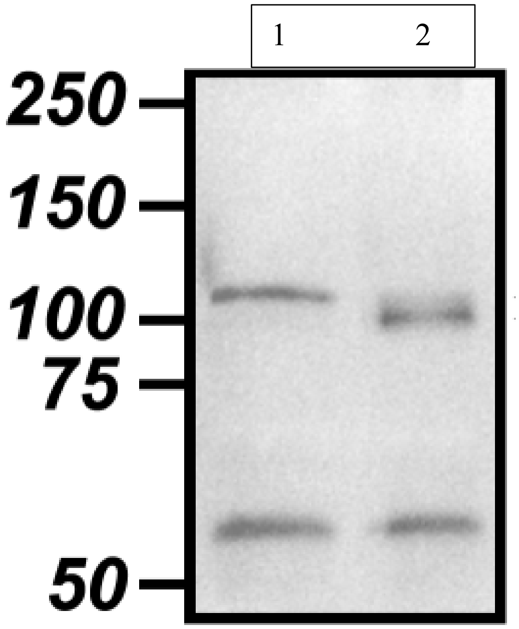

Band at 100 kDa	ROI Intensity
1	5711.104
2	6611.004

D) Blot for Supplementary Figure 2C

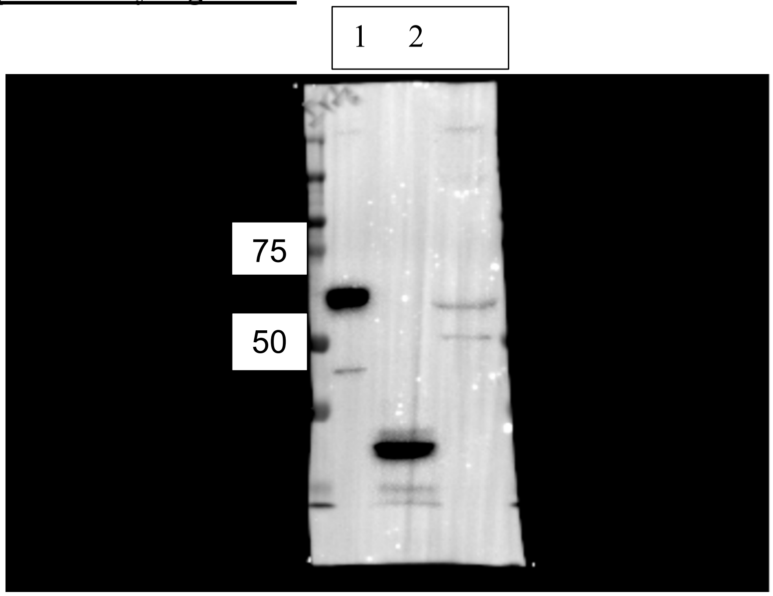

Band at 60 kDa	ROI Intensity
1	17494.723
2	151.778

5) Blot for Supplementary Figure 2D

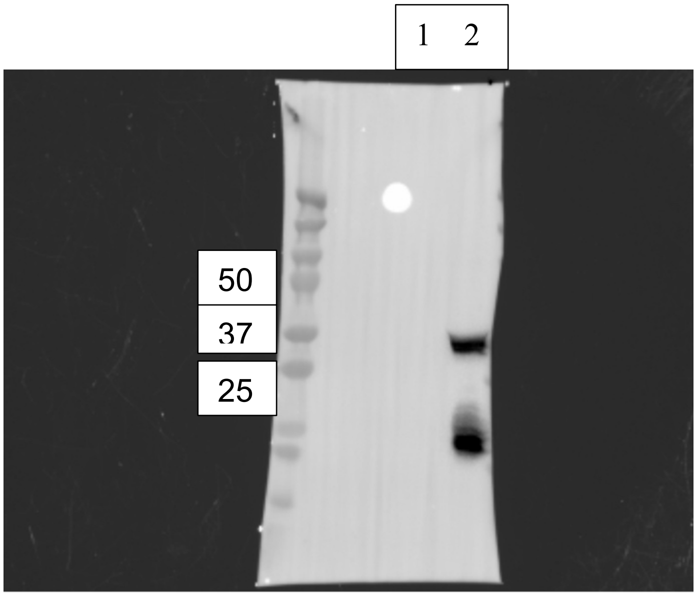

Band at 37 kDa	ROI Intensity
1	113.950
2	10735.401

Supplementary Figure 3: Original blots with densitometry performed using ImageJ.
